# Comparative Proteomics Analysis of Growth-Primed Adult Dorsal Root Ganglia Reveals Key Molecular Mediators for Peripheral Nerve Regeneration

**DOI:** 10.1523/ENEURO.0168-22.2022

**Published:** 2023-01-03

**Authors:** Maricris Bautista, George S. Katselis, Bari Chowdhury, Paulos Chumala, Ruhi Mahendra, Priyanshi Desai, Justin Hall, Subha Kalyaanamoorthy, Anand Krishnan

**Affiliations:** 1 Department of Anatomy, Physiology, and Pharmacology, College of Medicine, University of Saskatchewan, Saskatoon, Saskatchewan S7N 5E5, Canada; 2 Cameco Multiple Sclerosis Neuroscience Research Centre, Saskatoon, Saskatchewan S7K 0M7, Canada; 3 Canadian Centre for Health and Safety in Agriculture (CCHSA), Department of Medicine, College of Medicine, University of Saskatchewan, Saskatoon, Saskatchewan S7N 2Z4, Canada; 4 Department of Chemistry, University of Waterloo, Waterloo, Ontario N2L 3G1, Canada

**Keywords:** dorsal root ganglia, MANF, neurite outgrowth, peripheral nerve injury, priming, proteomics

## Abstract

Injuries to peripheral nerves are frequent, yet no drug therapies are available for effective nerve repair. The slow growth rate of axons and inadequate access to growth factors challenge natural repair of nerves. A better understanding of the molecules that can promote the rate of axon growth may reveal therapeutic opportunities. Molecular profiling of injured neurons at early intervals of injury, when regeneration is at the maximum, has been the gold standard for exploring growth promoters. A complementary *in vitro* regenerative priming model was recently shown to induce enhanced outgrowth in adult sensory neurons. In this work, we exploited the *in vitro* priming model to reveal novel candidates for adult nerve regeneration. We performed a whole-tissue proteomics analysis of the *in vitro* primed dorsal root ganglia (DRGs) from adult SD rats and compared their molecular profile with that of the *in vivo* primed, and control DRGs. The proteomics data generated are available via ProteomeXchange with identifier PXD031927. From the follow-up analysis, Bioinformatics interventions, and literature curation, we identified several molecules that were differentially expressed in the primed DRGs with a potential to modulate adult nerve regrowth. We then validated the growth promoting roles of mesencephalic astrocyte-derived neurotrophic factor (MANF), one of the hits we identified, in adult rat sensory neurons. Overall, in this study, we explored two growth priming paradigm and shortlisted several candidates, and validated MANF, as potential targets for adult nerve regeneration. We also demonstrate that the *in vitro* priming model is a valid tool for adult nerve regeneration studies.

## Significance Statement

Severe damages to peripheral nerves often lead to permanent functional disability. However, no pharmacological therapies are currently available for effective nerve repair because of the lack of knowledge of potent molecular targets. In this work, we performed whole-tissue proteomics studies using two growth priming models and revealed novel molecular candidates, including mesencephalic astrocyte-derived neurotrophic factor (MANF), for peripheral nerve regeneration. This work has not only generated a large list of potential molecular candidates for future research but also characterized a novel *in vitro* model at the molecular level for nerve regeneration studies.

## Introduction

Peripheral nerve injuries affect millions of people worldwide (Grinsell and Keating, 2014; López-Cebral et al., 2017). Severe injuries interrupt nerve continuity leading to long-term functional disabilities in affected individuals. Surgical reconstruction of injured nerves is a treatment choice for severe injuries. However, it does not often restore full functions because of the poor regeneration of axons (Bruyns et al., 2003; Bergmeister et al., 2020). For example, axons regenerate at a poor rate of 1–2 mm/d, which combined with insufficient access to growth factors, limits target reinnervation and functional recovery (Gordon, 2020). Approaches that can improve the rate of axon growth and availability of growth factors may improve nerve regeneration and functional recovery.

Several preclinical studies demonstrated that growth factors such as the nerve growth factor (NGF), brain-derived neurotrophic factor (BDNF) and glial-cell derived neurotrophic factor (GDNF) promote axon regeneration (Karchewski et al., 2002; Gordon, 2009). However, none of them translated into effective nerve repair therapies because of their poor efficacy or adverse outcomes in the clinical settings. This demands identification of novel molecular candidates with superior efficacies. A novel *in vitro* model, known as *in vitro* priming model, has been recently developed to study neuron outgrowth (Krishnan et al., 2021). This new model involves growth priming of sensory neurons by incubating the whole dorsal root ganglia (DRG) in suspension in a standard culture media *in vitro* overnight. It was demonstrated that the outgrowth capacity of the *in vitro* primed neurons is as good as the *in vivo* primed neurons, which are generated by a preconditioning transection injury to sciatic nerve (SN) in adult rats (Krishnan et al., 2021). However, the molecular profile of *in vitro* primed DRGs was not known. In this work, we characterized the *in vitro* primed DRGs using a whole-tissue proteomics approach and compared their molecular signature with that of the *in vivo* primed and nonprimed (control) DRGs. We found differential expression of several growth regulatory molecules in the *in vitro* and *in vivo* primed DRGs compared with control, revealing potential molecular targets for nerve regeneration and repair. Through this approach, we found a previously unrecognized growth promoting role for the mesencephalic astrocyte-derived neurotrophic factor (MANF) in the peripheral nervous system (PNS), along with the detection of its specific receptor, neuroplastin, in PNS tissues.

## Materials and Methods

### *In vitro* and *in vivo* priming of DRGs

*In vitro* and *in vivo* priming of L4–L6 DRGs were done as described previously (Krishnan et al., 2021). For the *in vitro* priming, intact DRGs from four- to six-week-old healthy male SD rats were incubated in standard primary neuron culture media [DMEM/F12 (catalog #11330032; ThermoFisher Scientific) + N2 supplement (catalog #17502048; ThermoFisher Scientific) + 100 ng/ml NGF (catalog #13257-019; ThermoFisher Scientific) + Cytiva Hyclone antibiotic/antimycotic solution (catalog #SV3007901; Fisher Scientific)] at 37°C and 5% CO_2_ conditions for 24 h. *In vivo* priming was done by performing axotomy in adult male SD rats. Briefly, a small incision was made at the mid-thigh level and muscle layers were separated using a fine scissor to expose sciatic nerve (SN). A blunt transection was then performed onto the SN for complete axotomy. The muscle layers and the skin were then sutured back, and the animals allowed to survive for 3 d before the isolation of DRGs. All animal protocols were approved by the Institutional Animal Research Ethics Board Committee.

### Whole-tissue proteomics studies

Total proteins from DRGs were isolated using RIPA lysis buffer (catalog #PI89900; Fisher Scientific) containing Halt protease and phosphatase inhibitor cocktail (catalog #PI78441; Fisher Scientific). Protein concentration was determined by Bradford assay. Proteins were denatured with trifluoroethanol (catalog #BP622-100; Fisher Scientific) and digested in-solution following a previously published protocol (Nair et al., 2021). Briefly, denatured proteins were reduced with dithiothreitol (catalog #194821; MP Biomedicals), alkylated with iodoacetamide (catalog #AC122270050; Fisher Scientific), and then treated with cold acetone (catalog #A18-4; Fisher Scientific) to eliminate interfering compounds (e.g., salts and detergents) that can prevent digestion. Proteins were digested overnight at 37°C in a buffer-containing trypsin (catalog #V5111; Promega Corporation; 50 ng/μl trypsin in hydrochloric acid)/ammonium bicarbonate) at a 40:1 protein:trypsin ratio. To ensure complete digestion of proteins into peptides, samples were further incubated the following morning for 2 h at 37°C in the same ratio trypsin buffer. Digested peptides were dried in speedvac (Labconco) and stored at −80°C until mass spectrometric analysis. Peptides were analyzed by liquid chromatography-tandem mass spectrometry (LC-MS/MS) using an Agilent 6550 iFunnel quadrupole time-of-flight mass spectrometer equipped with an Agilent 1260 series liquid chromatography instrument and a Chip Cube LC-MS interface (Agilent Technologies) in data dependent acquisition mode. Chromatographic peptide separation was accomplished using a high-capacity high performance LC Polaris chip (Polaris C18-A, 180 Å, 3-μm stationary phase; Agilent Technologies), consisting of a 360-nl enrichment column and a 75 μm × 150 mm analytical column. Peptide samples were first loaded onto the enrichment column with 0.1% formic acid in water (ThermoFisher Scientific) at a flow rate of 2.0 μl/min and then transferred onto the analytical column for gradient elution separation. The linear gradient system consisted of solvent A (0.1% formic acid in water) and solvent B [0.1% formic acid in acetonitrile (catalog #A9554; Fisher Scientific)]. The linear gradient program was 3–25% solvent B for 105 min followed by 25–90% solvent B for 20 min at a flow rate of 0.3 μl/min. Positive-ion electrospray MS data were acquired using a capillary voltage set at 1900 V, the ion fragmentor set at 360 V, and the drying/collision gas (nitrogen) set at 225°C with a flow rate of 12.0 l/min. Spectra were collected over a mass range of 250–1700 mass/charge (m/z) at a scan rate of 8 spectra/s. Tandem mass spectrometry data were collected over a range of 100–1700 m/z and a set isolation width of 1.3 atomic mass units. The top 20 most intense precursor ions for each MS scan were selected for MS/MS with active exclusion for 0.25 min.

Tandem mass spectra were searched against the SwissProt rat (*Rattus norvegicus*; UniProt May 2020 release date) nonredundant database using Spectrum Mill (Agilent Technologies) as the protein database search engine. Search parameters included a fragment mass error of 50 ppm, a parent mass error of 20 ppm, trypsin cleavage specificity, and carbamidomethyl as a fixed modification of cysteine. Carbamylated lysine, acetyl lysine, oxidized methionine, pyroglutamic acid, deamidated asparagine and phosphorylated serine, threonine, and tyrosine were used as variable modifications in various stages during the database search. In addition, semi-trypsin nonspecific C terminus and semi-trypsin nonspecific N terminus were also used during the search to enhance protein identification. Validation of the Spectrum Mill search was performed at peptide and protein levels (1% false discovery rate; FDR). Three samples each representing the control, *in vitro* primed and *in vivo* primed DRGs were used for the analysis. Differential expression of proteins in the *in vitro* and *in vivo* primed DRGs was determined relative to control based on a fold change (FC) of ≥2 in spectral intensities. Mass Profiler Professional (MPP, version 15.0, Agilent Technologies) software was used for the statistical analysis (one-way ANOVA). A cutoff value of *p* < 0.05 and the Benjamini and Hochberg FDR set at <1% were used to obtain statistically significant results. Each of the significant proteins has more than two peptides identified. The mass spectrometry proteomics data are deposited to the ProteomeXchange Consortium via the PRIDE partner repository with the dataset identifier PXD031927 (Perez-Riverol et al., 2022).

### Bioinformatics and functional enrichment analysis

The protein expression data as observed by the FC values were used to identify common and unique proteins differentially expressed in both *in vivo* and *in vitro* samples. The STRING database and the Gene Ontology (GO) terms were used to retrieve the biological processes for all differentially expressed proteins (Ashburner et al., 2000; Szklarczyk et al., 2021). In addition, for the commonly upregulated proteins (proteins that were commonly upregulated in both *in vivo* and *in vitro* samples compared with control), due their small number, we performed AmiGO2 gene ontology search for retrieving the biological functions (Carbon et al., 2009). Plots were generated using the ggplot library available in the R statistical package.

### Immunohistochemistry

Immunohistochemistry was performed according to the previously published protocol (Krishnan et al., 2018a). Briefly, the DRGs were fixed in Zamponi’s buffer overnight at 4°C, followed by cryoprotection in 20% sucrose overnight at 4°C. Tissue blocks were then made using optimal cutting temperature (OCT) compound and 12-μm tissue sections were collected on slides. For immunostaining, the sections were initially blocked using 5% donkey serum containing 0.3% Triton X-100. The primary antibodies used were, MANF (rabbit polyclonal, catalog #SAB3500384, Millipore Sigma, 1:50 dilution), NF200 (mouse monoclonal, catalog #N0142, Millipore Sigma, 1:200 dilution), GFAP (chicken polyclonal, catalog #PA1-1004, ThermoFisher Scientific, 1:200 dilution), and neuroplastin (rabbit polyclonal, catalog #PA5-77528, ThermoFisher Scientific, 1:200 dilution) for 1 h at room temperature. The secondary antibodies used were, goat anti-rabbit-Alexa Fluor 488 (catalog #A-11034; ThermoFisher Scientific; 1:100 dilution), goat anti-mouse-Alexa Fluor 647 (catalog #A-21235; ThermoFisher Scientific; 1:100 dilution), goat anti-mouse-Alexa Fluor 568 (catalog #A-11004; ThermoFisher Scientific; 1:100 dilution), and goat anti-chicken-Alexa Fluor 647 (catalog #A-21449; ThermoFisher Scientific; 1:100 dilution). The sections were then mounted using SlowFade Diamond Antifade Mountant with DAPI (catalog #S36973; ThermoFisher Scientific). The images were captured using an inverted fluorescence microscope (Zeiss Axio Observer).

### Neurite outgrowth analysis

Neurite outgrowth analysis was performed as described previously (Krishnan et al., 2018b). Briefly, adult DRG neurons at the thoracic and lumbar levels were isolated from six-week-old male SD rats. The DRGs were then incubated in 0.1% collagenase (catalog #17104019; ThermoFisher Scientific) in L15 (catalog #11415064; ThermoFisher Scientific) for 90 min at 37°C for enzymatic digestion. The individual cells were then mechanically dissociated from the DRGs by repeated pipetting, followed by the cells pelleted by centrifugation at 800 rpm for 6 min. The cell suspension was then laid over 15% bovine serum albumin (BSA; catalog #SH3057402; Fisher Scientific) and centrifuged at 800 rpm for 6 min to remove the debris that concentrated at the middle layer of the supernatant. The pellet was then washed with L15 and suspended in DMEM/F12 media. Equal number of cells were then seeded on a Nunc Lab-Tek 4 well Chamber Slide (catalog #177437; ThermoFisher Scientific) coated with 0.01% poly-l-lysine solution (catalog #A-005-C; Millipore Sigma) and 10 μg/ml laminin (catalog #23017015; ThermoFisher Scientific). The neurons were cultured in standard primary neuron culture media described above.

To examine the growth promoting effect of MANF, 100 ng/ml recombinant MANF (catalog #3748-MN; Biotechne) was supplemented to the culture for 48 h. The cultures were then fixed using 4% paraformaldehyde and stained with NF200 (mouse monoclonal, catalog #N0142, Millipore Sigma, 1:200 dilution) followed by incubation with the secondary antibody (goat anti-mouse-Alexa Fluor-488; catalog #A-11001; ThermoFisher Scientific; 1:100 dilution). The neurite outgrowth images were captured using the inverted fluorescence microscope (Zeiss Axio Observer) and the outgrowth parameters were measured using the WIS-Neuromath software (Rishal et al., 2013).

### Measurement of MANF in culture supernatants

We performed LC-MS/MS analysis in four individual sensory neuron cultures grown for 14 h to determine whether MANF is released by adult sensory neurons in culture. At first, total protein from culture supernatants was isolated using acetone precipitation protocol. Briefly, one part of the culture supernatant was added to 10 parts of acetone (1:10 ratio) and the mixture stored at −80°C overnight. The resulting supernatant was removed the next day, and 1:10 acetone was again added to the pellet and incubated at −80°C for another 1 h. The final supernatant was then removed and the pellet air dried. The sample pellet was dissolved in PBS before in-solution digestion, which was performed as described for the whole-tissue proteomics studies. Targeted MS analysis on tryptic peptides that resulted from *in silico* digestion of MANF was performed. Database search of the acquired spectra, for the identification of MANF, was done as described above.

## Results

### *In vitro* and *in vivo* priming induce common molecular changes in adult DRGs

**Figure 1.**
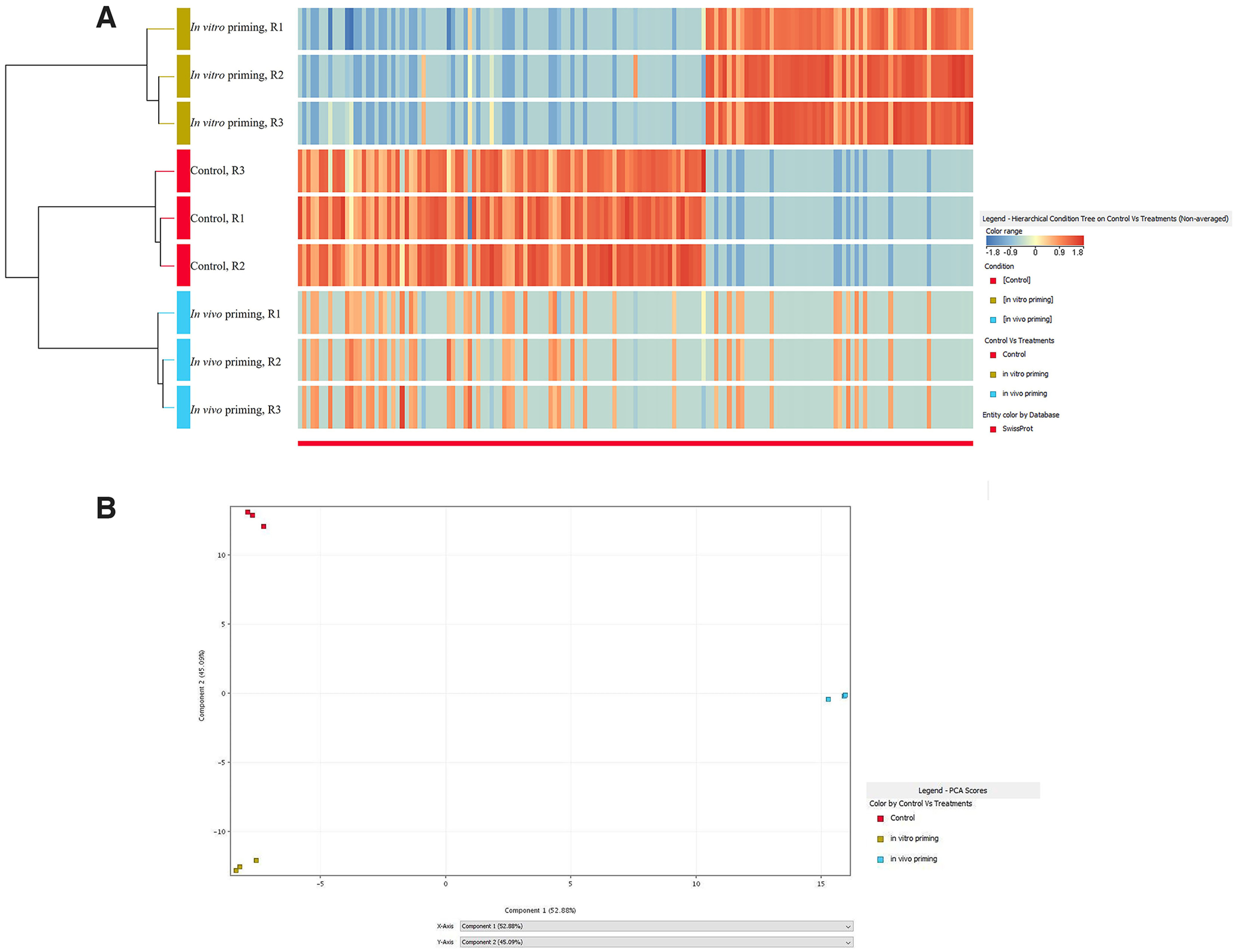
Heat map and principal component analysis (PCA) of the proteomics data. Heat map (***A***) and PCA (***B***) of the proteomics data shows similar protein profiles between the biological replicas of each group (see Extended Data Fig. 1-1 for the detailed presentation of the data with *p* values and fold change).

We detected a total of 1475 proteins in adult DRGs after processing the mass spectra data against the rat database (Extended Data Fig. 1-1). Principal component analysis (PCA) and heatmap showed a similar protein profile between the biological replicas (*n* = 3) for each group, indicating reproducibility in the results (Fig. 1).

10.1523/ENEURO.0168-22.2022.f1-1 Extended Data Figure 1-1 List of total proteins analyzed, and differentially expressed proteins (*p* ≤ 0.05) in the *in vivo* and *in vitro* primed DRGs are provided. Corrected *p* values and fold changes are also given. Potential functional roles of the proteins are also provided from the UniProt knowledgebase. Download Figure 1-1, XLS file.

Fold change analysis revealed that 63 and 64 proteins were upregulated in the *in vivo* and *in vitro* primed DRGs, respectively, compared with control (Extended Data Fig. 1-1). A single protein (coatomer subunit γ 2) was shown inverse differential expression (up and down) between the *in vivo* and *in vitro* models. Strikingly, 13 proteins were commonly upregulated in both the models indicating some shared molecular features in these models (Table 1). Similarly, 118 and 94 proteins were downregulated after *in vivo* and *in vitro* priming, respectively, compared with control (Extended Data Fig. 1-1). Out of these, 64 proteins were commonly downregulated in both models (Table 2). Overall, of the 263 differentially expressed proteins (*p* ≤0.05) observed combinedly in the two models, 77 (∼29%) proteins showed a similar change (up or down) between the models, indicating that *in vitro* priming partially reproduces the molecular features that occur in *in vivo* primed DRGs.

**Table 1:** List of common proteins upregulated (*p* ≤ 0.05) in the *in vivo* and *in vitro* primed DRGs and their potential functional roles from the UniProt knowledgebase

No	Protein	UniProt entry	Corresponding gene	Functional involvement
1	Methylglutaconyl-CoA hydratase, mitochondrial	F1LU71	*Auh*	Metabolism
2	Mesencephalic astrocyte-derived neurotrophic factor	P0C5H9	*MANF*	Neuron protection, neurite formation, ER stress regulation, growth factor
3	Pyruvate kinase PKLR	P12928	*PKLR*	Metabolism
4	Sarcoplasmic/endoplasmic reticulum calcium ATPase 3	P18596	*Atp2a3*	ATP hydrolysis, Calcium dynamics
5	NADH dehydrogenase [ubiquinone] flavoprotein 2, mitochondrial	P19234	*Ndufv2*	Electron transport
6	Coatomer subunit γ-1	Q4AEF8	*Copg1*	Protein transport
7	GPI transamidase component PIG-S	Q5XI31	*Pigs*	Glycosylphosphatidylinositol-anchor biosynthesis
8	F-actin-capping protein subunit β	Q5XI32	*Capzb*	Cytoskeleton organization
9	Ras-related protein Rap-1b	Q62636	*Rap1b*	Cell polarity (has intrinsic GTPase activity)
10	Plastin-3	Q63598	*Pls3*	Actin dynamics
11	Dolichyl-diphosphooligosaccharide--protein glycosyltransferase 48 kDa subunit	Q641Y0	*Ddost*	Protein glycosylation
12	Keratin, Type II cytoskeletal 75	Q6IG05	*Krt75*	Component of keratin intermediate filament
13	Electron transfer flavoprotein-ubiquinone oxidoreductase, mitochondrial	Q6UPE1	*Etfdh*	Electron transport chain, response to oxidative stress.

**Table 2:** List of common proteins downregulated (*p* ≤ 0.05) in the *in vivo* and *in vitro* primed DRGs and their potential functional roles from the UniProt knowledgebase

No.	Protein	UniProt entry	Corresponding gene	Functional involvement
1	Eukaryotic translation initiation factor 3 subunit I	B0BNA7	*Eif3i*	mRNA processing
2	Neutral cholesterol ester hydrolase 1	B2GV54	*Nceh1*	Lipid metabolism
3	Vacuolar protein sorting-associated protein 29	B2RZ78	*Vps29*	Protein transport
4	Filamin-C	D3ZHA0	*Flnc*	Actin binding
5	Brefeldin A-inhibited guanine nucleotide-exchange protein 1	D4A631	*Arfgef1*	Guanyl-nucleotide exchange, Golgi organization, neuron projection, cell polarity
6	RNA 5′-monophosphate methyltransferase	D4ABH7	*Bcdin3d*	RNA regulation
7	Kinesin-like protein KIF1A	F1M4A4	*Kif1a*	Vesicle transport
8	Glycogenin-1	O08730	*Gyg1*	Glycogen biosynthesis
9	Breast cancer type 2 susceptibility protein homolog	O35923	*Brca2*	DNA repair
10	Xaa-Pro aminopeptidase 1	O54975	*Xpnpep1*	Bradykinin catabolism, proteolysis
11	Acyl-coenzyme A thioesterase 1	O88267	*Acot1*	Fatty acid metabolism
12	Transthyretin	P02767	*Ttr*	Retinol metabolic process, hormone transport
13	Collagen α-1(II) chain	P05539	*Col2a1*	Extracellular matrix component
14	Protein kinase C α type	P05696	*Prkca*	Cell proliferation, migration, cell adhesion
15	Ras-related protein Rab-2A	P05712	*Rab2a*	Protein transport
16	Medium-chain specific acyl-CoA dehydrogenase, mitochondrial	P08503	*Acadm*	Lipid metabolism
17	Sarcoplasmic/endoplasmic reticulum calcium ATPase 2	P11507	*Atp2a2*	Calcium homeostasis
18	cAMP-dependent protein kinase Type II-β regulatory subunit	P12369	*Prkar2b*	Regulation of protein kinase activity
19	Neural cell adhesion molecule 1	P13596	*Ncam1*	Cell adhesion, neuron projection, axon regeneration
20	Aldehyde dehydrogenase, cytosolic 1	P13601	*Aldh1a7*	Oxidoreductase
21	L-lactate dehydrogenase C chain	P19629	*Ldhc*	Metabolism
22	Platelet-derived growth factor receptor α	P20786	*Pdgfra*	Growth factor receptor
23	60S ribosomal protein L3	P21531	*Rpl3*	Translational regulation
24	Macrophage migration inhibitory factor	P30904	*Mif*	Pro-inflammatory cytokine
25	Proteasome subunit β type-4	P34067	*Psmb4*	Protein catabolism
26	Proteasome subunit β type-3	P40112	*Psmb3*	Protein catabolism
27	Signal peptidase complex catalytic subunit SEC11A	P42667	*Sec11a*	Signal peptide processing
28	Cysteine and glycine-rich protein 1	P47875	*Csrp1*	Actin cytoskeleton organization
29	Glycogen phosphorylase, brain form (Fragment)	P53534	*Pygb*	Glycogen metabolism
30	α-Soluble NSF attachment protein	P54921	*Napa*	Vesicle transport
31	Vesicle transport through interaction with t-SNAREs homolog 1B	P58200	*Vti1b*	Vesicle transport
32	Dihydrolipoyllysine-residue succinyltransferase component of 2-oxoglutarate dehydrogenase complex, mitochondrial	Q01205	*Dlst*	Metabolism
33	Cyclin-dependent-like kinase 5	Q03114	*Cdk5*	Kinase activity, cell division, neuron projection
34	Drebrin	Q07266	*Dbn1*	Cytoskeletal organization (actin)
35	Piwi-like protein 4	Q4G033	*Piwil4*	piRNA binding
36	UMP-CMP kinase	Q4KM73	*Cmpk1*	Nucleotide biosynthesis
37	AH receptor-interacting protein	Q5FWY5	*Aip*	Aryl-hydrocarbon receptor binding
38	Endophilin-B2	Q5PPJ9	*Sh3glb2*	Cadherin binding, EB2 plays an indispensable role in mitochondria sequestration and inner mitochondrial membrane (IMM) protein degradation during mitophagy.
39	Aldehyde oxidase 2	Q5QE78	*Aox2*	Oxidoreductase, metabolism
40	Membrane-associated progesterone receptor component 2	Q5XIU9	*Pgrmc2*	Nonclassical progesterone receptor
41	Synaptojanin-1	Q62910	*Synj1*	Phosphatase
42	Diphosphomevalonate decarboxylase	Q62967	*Mvd*	Cholesterol biosynthesis
43	Contactin-1	Q63198	*Cntn1*	Cell adhesion, notch signaling
44	Proteasome activator complex subunit 1	Q63797	*Psme1*	Endopeptidase activity, regulation of protein catabolism
45	Probable ATP-dependent RNA helicase DDX4	Q64060	*Ddx4*	RNA helicase activity
46	Peroxisomal membrane protein PEX14	Q642G4	*Pex14*	Peroxisome transport
47	Calcium-transporting ATPase type 2C member 1	Q64566	*Atp2c1*	Calcium and manganese transporter activity
48	Plasma membrane calcium-transporting ATPase 3	Q64568	*Atp2b3*	Calcium transport
49	Solute carrier family 22 member 18	Q6AY78	*Slc22a18*	Ion transport
50	Keratin, Type I cytoskeletal 13	Q6IFV4	*Krt13*	Cytoskeleton organization, structural molecule
51	Tryptophan--tRNA ligase, cytoplasmic	Q6P7B0	*Wars1*	Regulates ERK, Akt and eNOS activation pathways
52	Cytosolic nonspecific dipeptidase	Q6Q0N1	*Cndp2*	Proteolysis
53	N-α-acetyltransferase 25, NatB auxiliary subunit	Q6QI44	*Naa25*	Peptidyl methionine acetylation
54	Golgi resident protein GCP60	Q7TNY6	*Acbd3*	Fatty acyl-coA binding, steroid biosynthetic process
55	MAP/microtubule affinity-regulating kinase 3	Q8VH	*Mark3*	Kinase, microtubule organization
56	Vascular endothelial growth factor receptor 3	Q91ZT1	*Flt4*	Growth factor receptor
57	Long-chain-fatty-acid--CoA ligase ACSBG1	Q924N5	*Acsbg1*	Fatty acid metabolism
58	Lactosylceramide 1,3-N-acetyl-β-D-glucosaminyltransferase	Q99NB2	*B3gnt5*	Protein glycosylation
59	Otoferlin	Q9ERC5	*Otof*	Calcium binding, synaptic vesicle excocytosis
60	DNA damage-binding protein 1	Q9ESW0	*Ddb1*	DNA damage response
61	Apoptosis-inducing factor 1, mitochondrial	Q9JM53	*Aifm1*	NADH oxidoreductase, regulator of apoptosis
62	NADH dehydrogenase [ubiquinone] 1 α subcomplex assembly factor 4	Q9NQR8	*Ndufaf4*	Positive regulation of cell proliferation
63	ProSAAS	Q9QXU9	*Pcsk1n*	Neuropeptide signaling pathway, control neuroendocrine secretory pathway
64	Peroxisomal biogenesis factor 19	Q9QYU1	*Pex19*	Chaperone

### *In vivo* and *in vitro* primed adult DRGs upregulate common proteins involved with metabolism, neuron survival, and outgrowth

**Figure 2.**
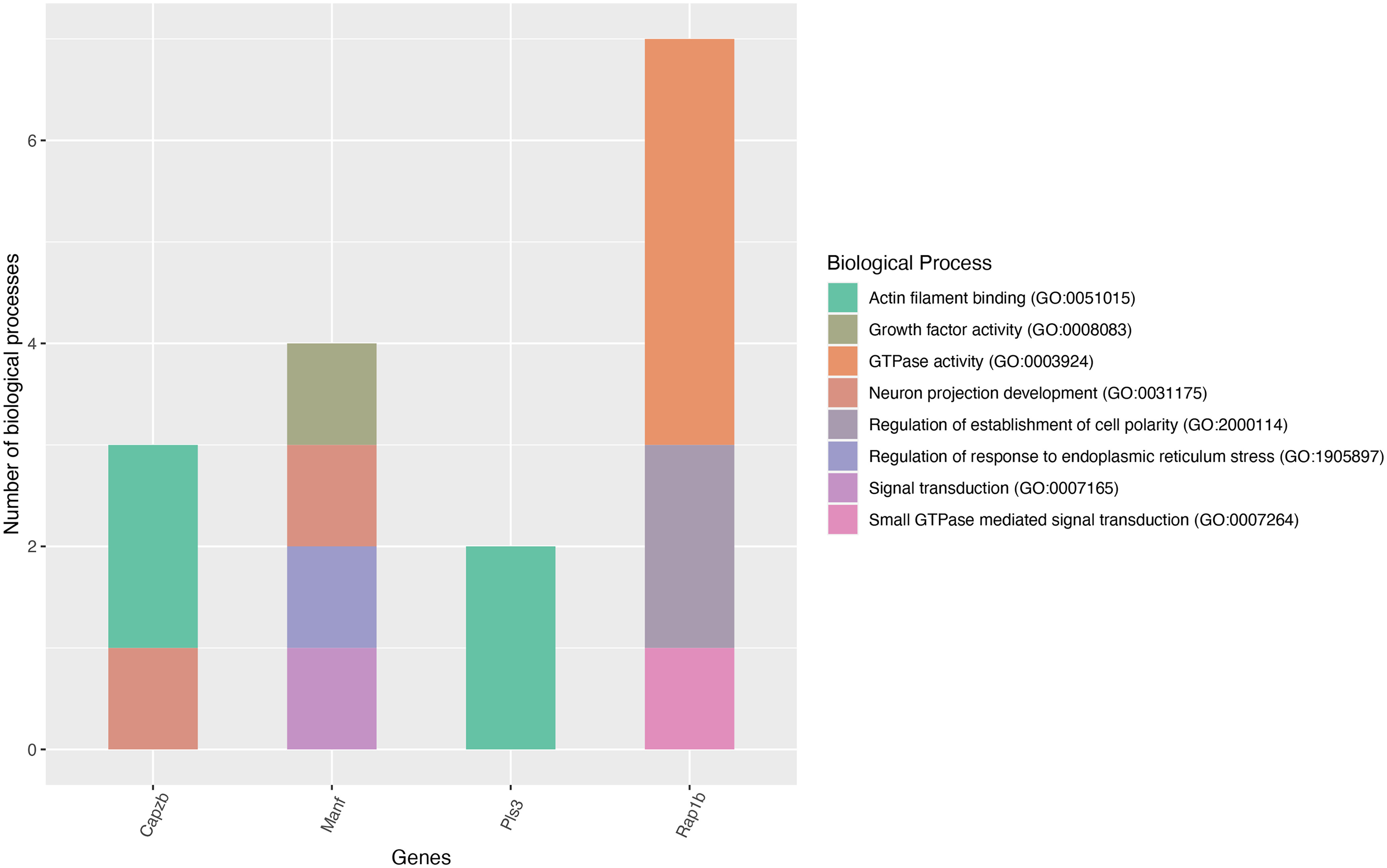
Nerve growth-related biological processes of the commonly upregulated proteins. Plot showing the potential nerve growth-related functions of commonly upregulated proteins in the *in vivo* and *in vitro* primed DRGs (gene names are given on the plot). AMIGO gene ontology search was performed for the functional enrichment analysis (see Extended Data Fig. 2-1 for additional details). The plot was generated using ggplot library in the R statistical package.

We found that 13 proteins were commonly upregulated in the *in vitro* and *in vivo* primed DRGs (Table 1). UniProt knowledgebase indicated that these proteins are associated with cell metabolism, transport, cytoskeleton organization and dynamics, stress regulation, and neuron projection and survival functions (Table 1). Increased metabolism, stress related signaling cascade, neurite outgrowth and structural reorganization are critical events for efficient nerve regeneration. Hence, it is interesting to note that *in vitro* priming also induces such molecular changes in DRGs in similar ways as observed after *in vivo* priming. Because of the small number of proteins in this pool, we used AmiGO2 gene ontology search for their functional enrichment analysis (Extended Data Fig. 2-1). Following, our focused analysis identified the candidates that enrich nerve growth-related biological processes, such as, actin filament binding, growth factor activity, GTPase activity, neuron projection development, cell polarity establishment, response to endoplasmic reticulum stress, and signal transduction (Fig. 2). Strikingly, we noted that MANF and Rap-1b enrich at least three biological processes related to nerve growth (Fig. 2). MANF and Rap-1b were previously shown to promote the survival, polarization and outgrowth of the CNS neurons (Schwamborn and Püschel, 2004; Tseng et al., 2017; Eesmaa et al., 2021). However, their growth modulatory roles in the PNS neurons are not well established. We also found that plastin-3 is commonly upregulated in the primed DRGs. Plastin-3 was earlier shown to promote CNS axon growth and survival (Alrafiah et al., 2018). Overall, we found that *in vitro* priming induces at least some critical molecular changes in adult DRGs similar to what seen after *in vivo* priming, suggesting that *in vitro* primed DRG may be an additional suitable model for exploring potential molecular cues involved with adult PNS regeneration.

10.1523/ENEURO.0168-22.2022.f2-1 Extended Data Figure 2-1 Functional enrichment of the commonly upregulated proteins determined using AMIGO gene ontology search. Download Figure 2-1, XLS file.

### *In vivo* and *in vitro* primed adult DRGs downregulate common proteins that modulate biological processes related to nerve regeneration

**Figure 3.**
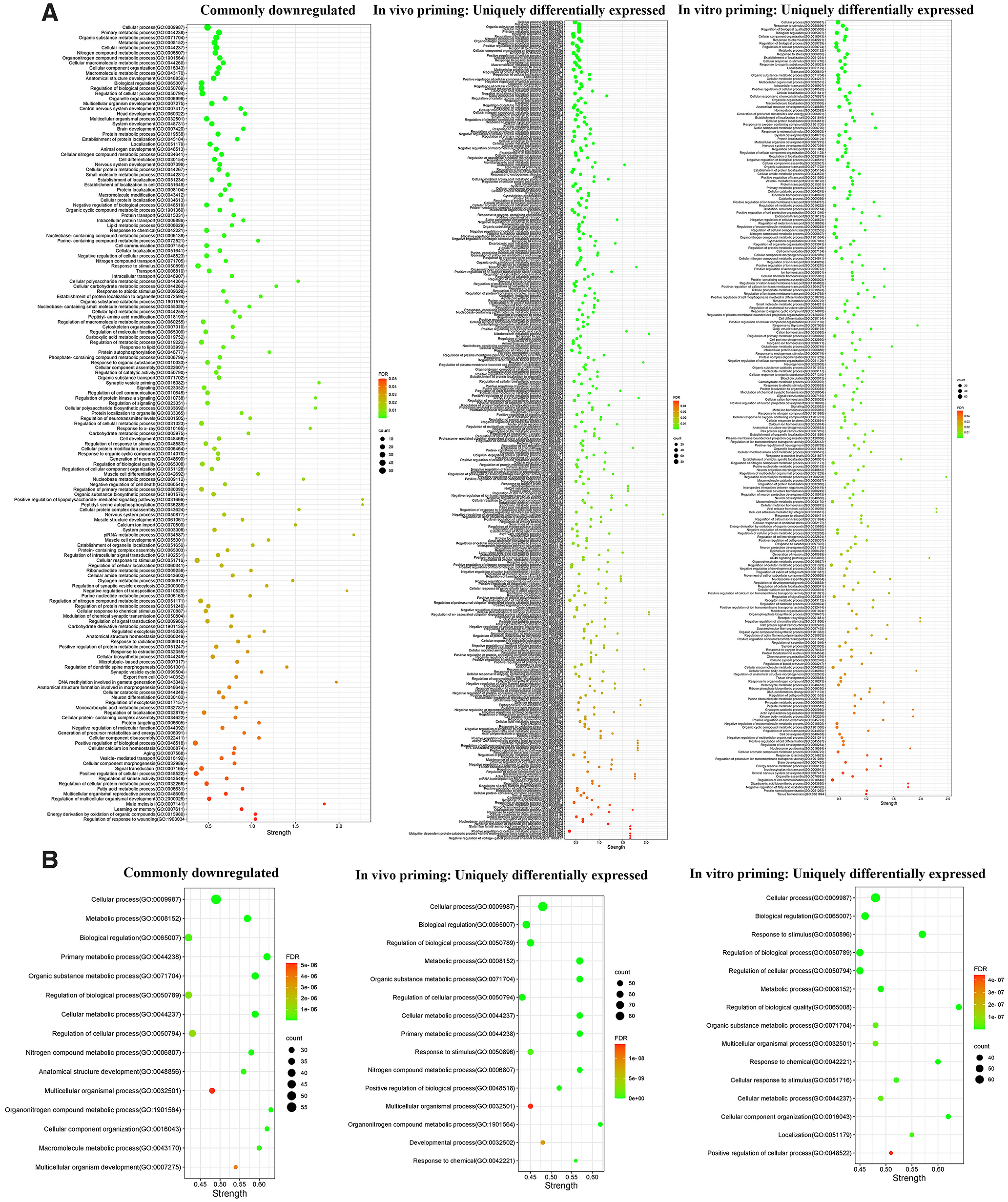
Functional enrichment analysis of differentially expressed proteins. ***A***, Functional enrichment analysis of the commonly downregulated and uniquely differentially expressed proteins in the *in vivo* and *in vitro* primed DRGs using the STRING database shows enriched biological processes. ***B***, Top 15 biological processes ranked by protein counts and strength.

**Figure 4.**
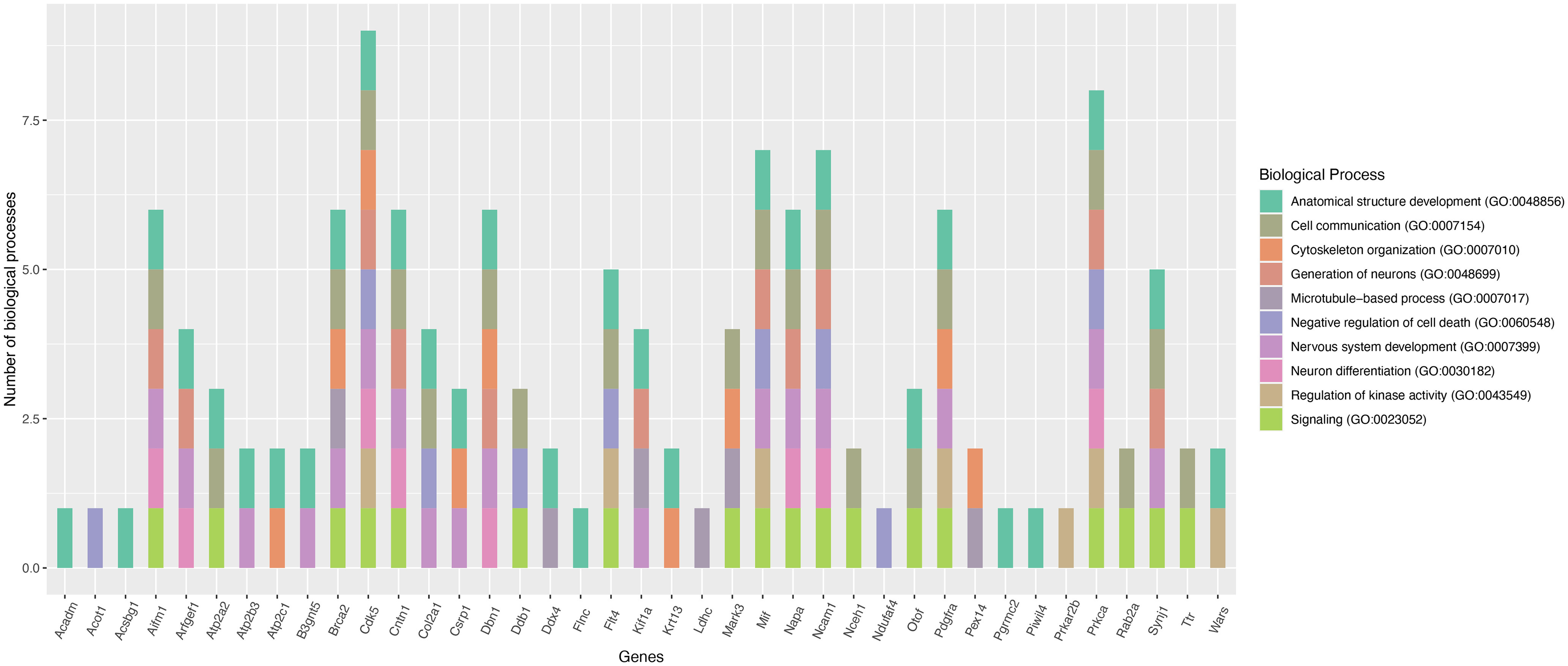
Nerve growth-related biological processes of the commonly downregulated proteins. Plot showing the potential nerve growth-related functions of commonly downregulated proteins the *in vivo* and *in vitro* primed DRGs (gene names are given on the plot). STRING database was used for the functional enrichment analysis. The plot was generated using ggplot library in the R statistical package.

We found that 64 common proteins were downregulated in the two primed DRG models compared with control. The list of commonly downregulated proteins and their molecular functions identified from the UniProt knowledgebase are given in Table 2. We also performed functional enrichment analysis of these proteins using the STRING database and found them clustering at several biological processes, including cellular and metabolic processes, biological regulation, structure development, and cellular component organization, etc. (Fig. 3). Our focused analysis then identified proteins that clustered onto the biological processes related to nerve regeneration, such as, anatomic structure development, nervous system development, cell communication, cytoskeleton organization, signaling, neurogenesis, microtubule-based process, neuron differentiation, kinase activity and negative regulation of cell death. This focused analysis revealed 39 potential proteins for future investigation (Fig. 4). Some of these proteins, such as, cyclin-dependent kinase 5 (*cdk5*), neural cell adhesion molecule 1 (*Ncam1*), and macrophage migration inhibitory factor (*Mif*) were previously shown to have growth regulatory roles in the PNS, while drebrin (*Dbn1*) and contactin-1 (*Cntn1*) were known to regulate CNS neurons (Nishio et al., 2002; Thornton et al., 2008; Tanabe et al., 2014; Koganezawa et al., 2017; Y.A. Chen et al., 2018; Hwang and Namgung, 2020). Considering the growth promoting roles of these proteins, it is likely that their supplementation may be therapeutic for nerve regeneration. Capturing these common molecular changes in the *in vitro* and *in vivo* primed DRGs indeed substantiates the validity of the *in vitro* priming model for future studies. Interestingly, the common downregulation of the transport proteins, the kinesin-like protein KIF1A (*Kif1a*), α-soluble NSF attachment protein (*napa*) and plasma membrane calcium-transporting ATPase 3 (*Atp2b3*) suggest that these proteins may not be critical for the communication of injury signals or transport of growth-related molecules across injured nerve tips and neuronal soma during regenerative reprogramming of adult neurons. Similarly, common downregulation of the actin modulators such as filamin-C and brefeldin A-inhibited guanine nucleotide-exchange protein 1 (*Arfgef1*) suggests that similar modes of cytoskeletal changes occur in neurons, and may be satellite glial cells, in the *in vitro* and *in vivo* primed DRGs. Although we found downregulation of a few growth promoters in the two models, additional molecular changes unique to these individual models may compensate the loss of these proteins for promoting outgrowth in the primed neurons. The unique protein changes observed in the two priming models are presented below.

### Unique molecular changes in the *in vivo* primed adult DRGs

**Figure 5.**
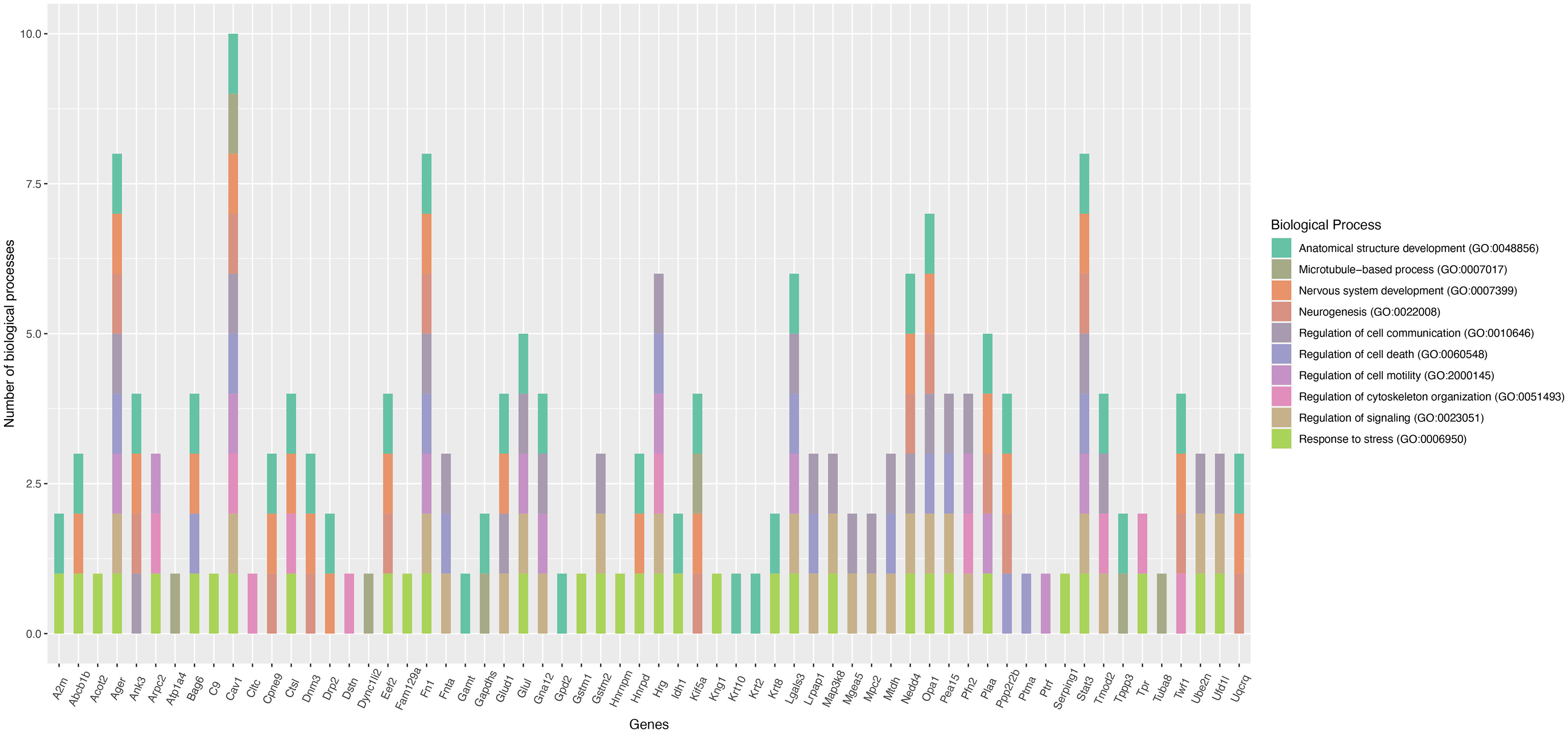
Nerve growth-related biological processes of the uniquely differentially expressed proteins in the *in vivo* primed DRGs. Plot showing the potential nerve growth-related functions of uniquely expressed proteins in the *in vivo* primed DRGs (gene names are given on the plot). STRING software was used for the functional enrichment analysis. The plot was generated using ggplot library in the R statistical package.

Expression of 104 proteins was uniquely altered in *in vivo* primed DRGs with 50 and 54 proteins upregulated and downregulated, respectively, compared with control. The list of these proteins and their potential functions identified from the UniProt knowledgebase are given in Extended Data Figure 1-1. STRING database-based functional enrichment analysis clustered them at the biological processes, including cellular and metabolic processes, response to chemical, response to stimulus, and development process, etc. (Fig. 3). Further, our specific focus on nerve growth-related biological processes, such as, anatomic structure development, microtubule-based process, nervous system development, neurogenesis, regulation of cell communication, regulation of cell death, regulation of cell motility, regulation of cytoskeleton organization, regulation of signaling, and response to stress revealed 62 potential proteins for future investigation (Fig. 5). Some of these proteins, such as, fibronectin (*Fn1*), signal transducer and activator of transcription 3 (*Stat3*) and E3 ubiquitin-protein ligase NEDD4, (*Nedd4*) are well known for their roles in PNS regeneration indicating the validity of our proteomics analysis (Bailey et al., 1993; Bareyre et al., 2011; Christie et al., 2012). The upregulation of the kinase, the mitogen activated protein kinase kinase kinase 8 (*Map3k8*), in this model suggests its potential role in promoting neurite extension. Similarly, the upregulation of the transport proteins such as dynamin-3 (*Dnm3*), twinfilin-1 (*Twf1*), cytoplasmic dynein 1 light intermediate chain 2 (*Dync1li2*), sodium/potassium-transporting ATPase subunit α−4 (*Atp1a4*), tubulin α−8 chain (*Tuba8*), and kinesin heavy chain isoform 5A (*Kif5a*) indicates their likely roles in communicating injury signals, and transporting growth promoting molecules across the injured nerve tips and neuronal soma, during the regenerative reprogramming of adult PNS neurons. Overall, our proteomics data substantiated that *in vivo* priming promotes key events such as cell metabolism, intracellular transport and trophic signaling cascades to facilitate neuron outgrowth and revealed additional molecular candidates for future exploration in adult nerve regeneration and repair models.

We found that a few proteins that modulate actin dynamics were downregulated in the *in vivo* primed DRGs (Extended Data Fig. 1-1). These were tropomodulin-2 (*Tmod2*), actin-related protein 2/3 complex subunit 2 (*Arpc2*), destrin (*Dstn*), and profilin-2 (*Pfn2*). The Tubulin polymerization-promoting protein family member 3 (*Tppp3*), which regulates tubulin dynamics, was also downregulated. We believe that this is because of *in vivo* priming favoring major cytoskeletal reorganization, a key prerequisite for neurite elongation. We found that both actin polymerizing (*Arpc2*) and depolymerizing (*Dstn*) proteins were downregulated indicating a balanced scenario. We also noted that the neurite outgrowth promoters, such as, caveolin-1 (*Cav1*) and elongation factor 2 (*Eef2*) were also downregulated after *in vivo* priming (Iketani et al., 2013; Wang et al., 2019). How their alterations influence the overall outgrowth of the *in vivo* primed neurons is unknown to us at present and may need further investigation.

### Unique molecular changes in the *in vitro* primed adult DRGs

**Figure 6.**
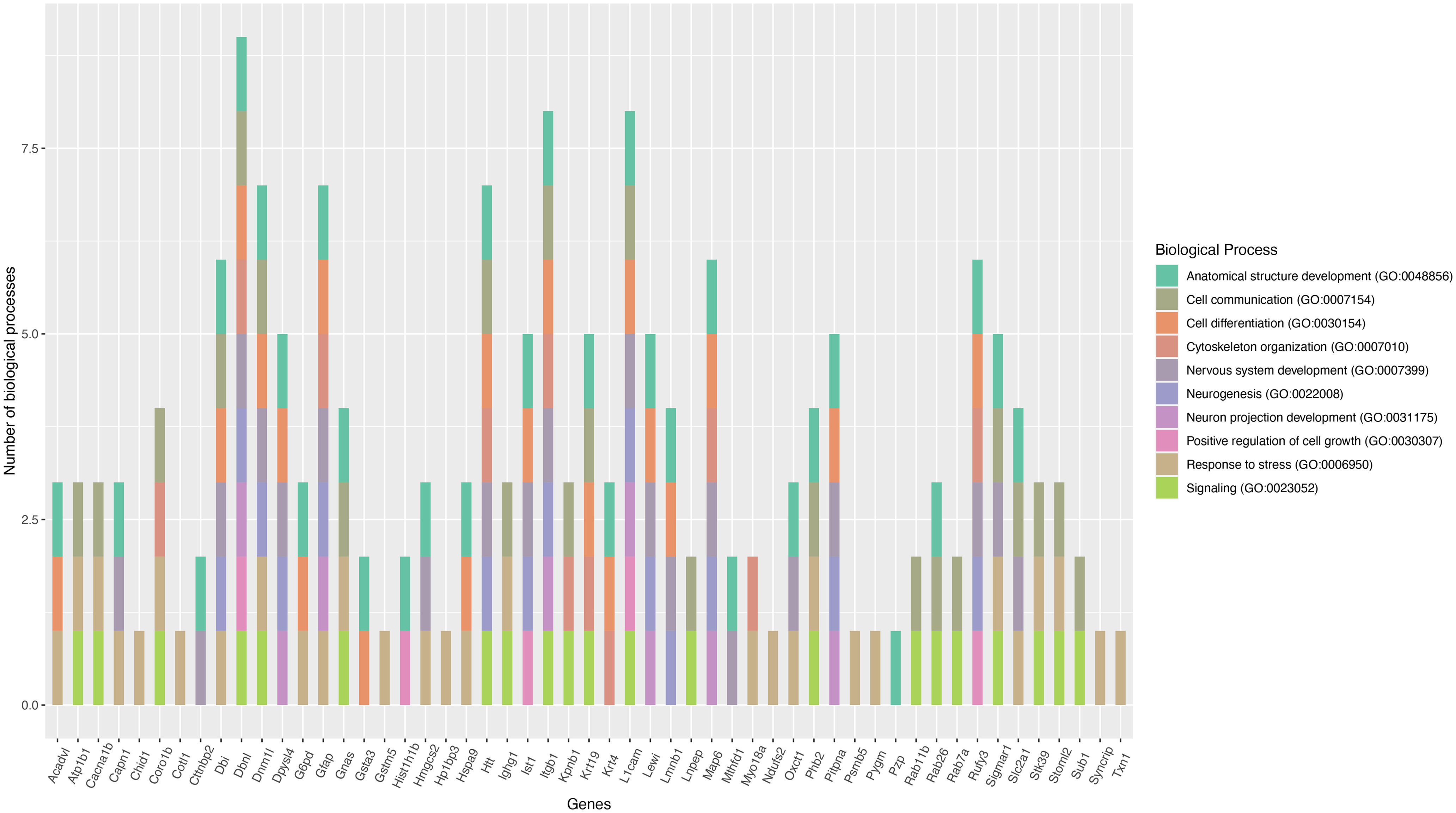
Nerve growth-related biological processes of the uniquely differentially expressed proteins in the *in vitro* primed DRGs. Plot showing the potential nerve growth-related functions of uniquely expressed proteins in the *in vitro* primed DRGs (gene names are given on the plot). STRING software was used for the functional enrichment analysis. The plot was generated using ggplot library in the R statistical package.

Expression of 81 proteins was uniquely altered in the *in vitro* primed DRGs with 51 and 30 proteins upregulated and downregulated, respectively, compared with control. The list of upregulated and downregulated proteins and their associated individual molecular functions identified from the UniProt database are given in Extended Data Figure 1-1. STRING database-based functional enrichment analysis clustered most of these proteins at the biological processes, such as, cellular and metabolic processes, response to stimulus, cellular component organization, response to chemical, response to stress, and establishment of localization, etc. (Fig. 3). Further, our specific focus on nerve growth-related biological processes, such as, anatomic structure development, cell communication, cell differentiation, cytoskeleton organization, nervous system development, neurogenesis, neuron projection development, positive regulation of cell growth, response to stress and signaling revealed 53 potential proteins for future investigation (Fig. 6). The upregulated proteins in this model were mainly associated with actin binding, intracellular transport, and transcriptional regulation (Extended Data Fig. 1-1). For example, the actin binding proteins, such as, coactosin-like protein (*Cotl1*) and coronin-1B (*Coro1b*) were upregulated. Similarly, the transport proteins sodium/potassium-transporting ATPase subunit β−1 (*Atp1b1*), solute carrier family 2 facilitated glucose transporter member 1 (*Slc2a1*), and importin subunit β−1 (*Kpnb1*) were upregulated. Among the transcriptional regulators, prohibitin-2 (*Phb2*) and activated RNA polymerase II transcriptional coactivator p15 (*Sub1*) were upregulated. Transcriptional activation and actin-dependent cytoskeletal modifications are critical events for peripheral nerve regeneration. Similarly, molecular transport between Golgi and endoplasmic reticulum, and nuclear and cytoplasmic compartments is also critical for efficient nerve regrowth. We also found that the axon growth promoter integrin β−1 (*Itgb1*) and the dendritic spine maintenance protein cortactin-binding protein 2 (*Cttnbp2*) were upregulated in the DRGs after *in vitro* priming (Y.K. Chen and Hsueh, 2012; Nieuwenhuis et al., 2018). The upregulation of these proteins might contribute to the enhanced outgrowth of the *in vitro* primed neurons and substantiates the validity of this model for future studies addressing axon regeneration.

We found that the growth modulators, such as, microtubule-associated protein 6 (*Map6*), protein RUFY3 (*Rufy3*) and neural cell adhesion molecule L1 (*L1cam*) were downregulated in the *in vitro* primed DRGs (Extended Data Fig. 1-1). However, their exact roles in adult peripheral nerve regeneration are not clearly understood and need additional investigation.

### Validation of the expression and growth promoting role of MANF in adult PNS neurons

**Figure 7.**
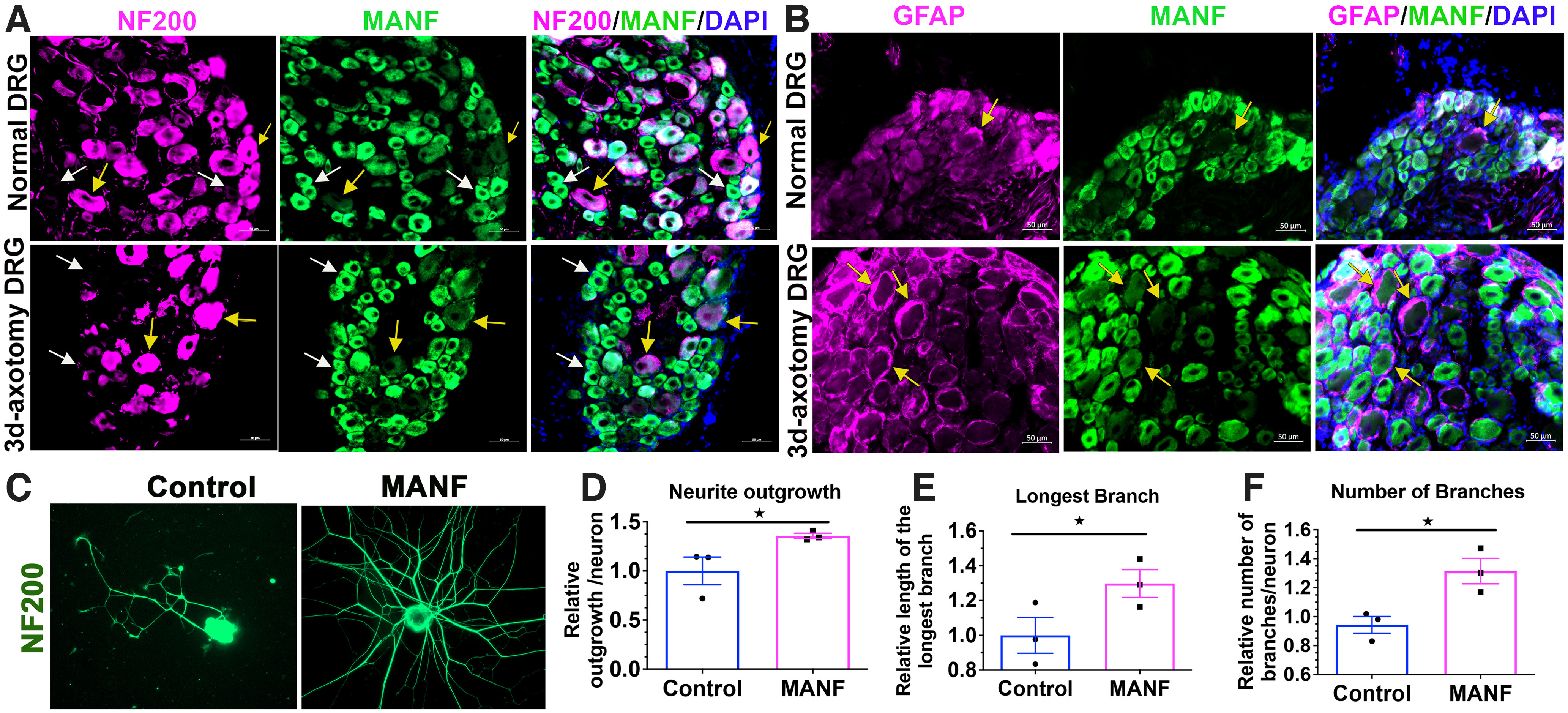
Validation of expression and growth promoting roles of MANF in peripheral neurons. ***A***, Immunostaining of normal and injured DRGs show MANF expression mostly in NF200^low^ sensory neurons (white arrows show NF200^low^/MANF^high^ neurons, while yellow arrows show NF200^high^/MANF^low^ neurons). ***B***, MANF shows very low to no expression in satellite glial cells (SGCs; stained using GFAP) in adult rat DRGs. Representative positions of SGCs are shown by yellow arrows. ***C–F***, MANF (100 ng/ml for 48 h) improves overall neurite outgrowth, branch length and number of branches in adult primary sensory neuron cultures (*n* = 3; mean ± SEM; *p < 0.05; Student’s *t* test).

We next validated the expression and growth promoting roles of the commonly upregulated protein MANF in adult PNS neurons. We were particularly interested in MANF because of its known neuroprotective and growth promoting roles in CNS neurons (Tseng et al., 2017; Eesmaa et al., 2021). However, its role in PNS neurons was not established. We examined its basal expression in adult primary sensory neurons from rats and found that it is expressed at moderate to high levels in NF200^low^ small caliber neurons and lacked expression in most of the NF200^high^ large caliber neurons (Fig. 7*A*, upper panel). Three-day injury to sciatic nerve increased the expression of MANF in NF200^low^ small caliber neurons, but strikingly, most of the NF200^high^ neurons still had no or lower MANF expression (Fig. 7*A*, lower panel). Given the known neurotrophic and neuroprotective actions of MANF in CNS neurons, we speculated that supplementation of MANF, especially to NF200^high^ large caliber neurons, may improve neurite outgrowth. To examine this, we supplemented MANF (100 ng/ml) in adult primary sensory neuron cultures and found that it promotes neurite outgrowth, especially in NF200^high^ neurons, significantly (Fig. 7*C*,*D*). MANF also improved the number of branches and branch length in adult neurons substantiating its growth promoting roles in the PNS (Fig. 7*E*,*F*).

We found very low to almost no detectable expression of MANF in satellite glial cells, especially after sciatic nerve injury (Fig. 7*B*). Overall, the validation of the expression of MANF in injured DRGs, and demonstration of its novel growth promoting role in the PNS, indicate the promise of the *in vitro* priming model as an ideal tool for exploring novel growth promoters in the PNS.

We also asked whether MANF is secreted by adult neurons in our culture conditions. To address this, we performed LC-MS/MS-based detection of rat MANF in the supernatants of adult rat primary sensory neuron cultures grown for 14 h. We found no detectable levels of MANF in the supernatant (data not shown), indicating that no significant release of MANF occurs in the culture conditions we employed.

### The MANF receptor neuroplastin is expressed in the PNS

**Figure 8.**
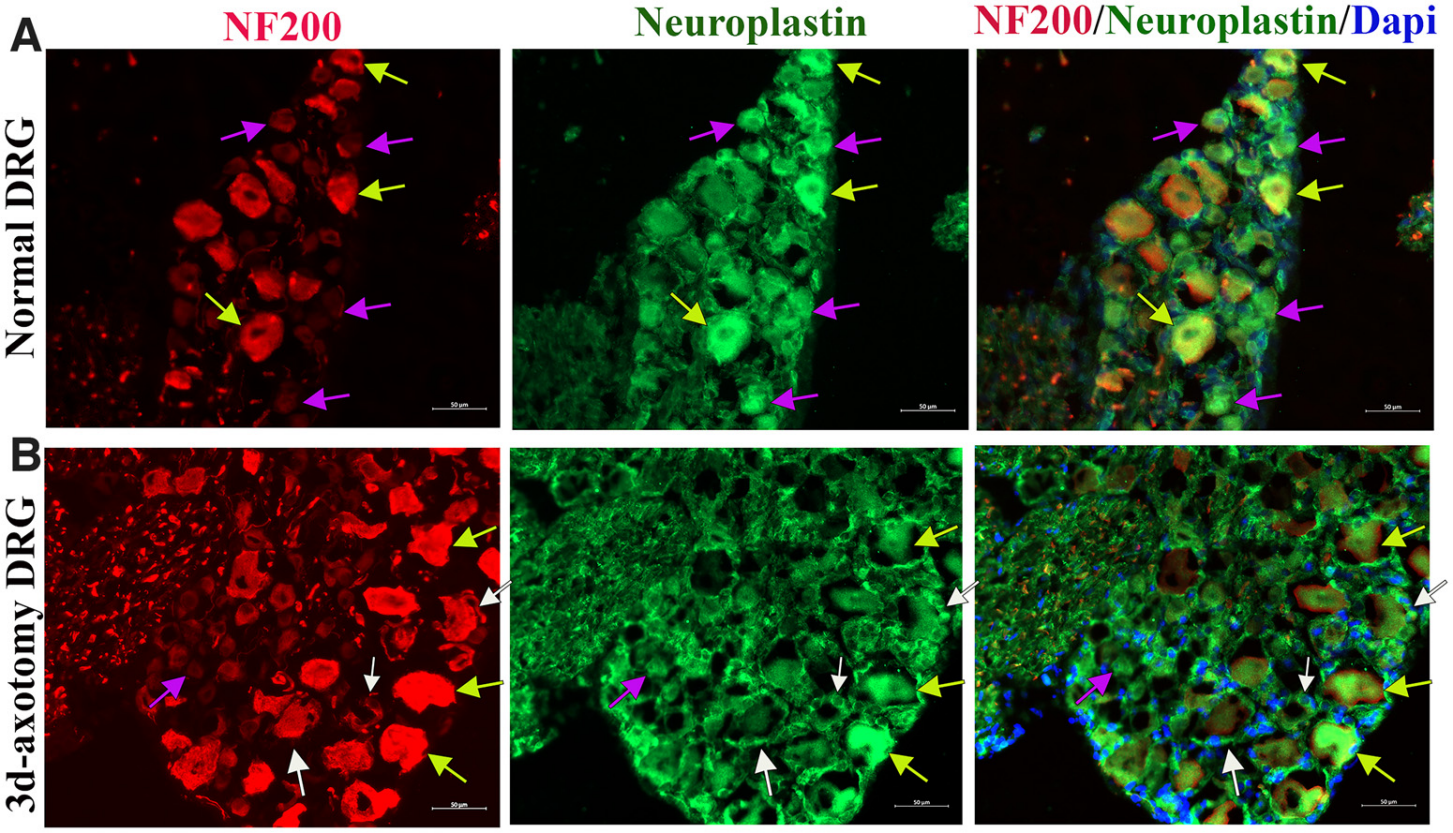
Neuroplastin expression in normal and 3-d axotomized DRGs. ***A***, Immunostaining of normal DRG shows the expression of neuroplastin in NF200^low^ (purple arrows) and NF200^high^ (yellow arrows) sensory neurons. ***B***, Immunostaining of 3-d axotomized DRG shows the expression of neuroplastin in NF200^low^ (purple arrow; mild expression) and NF200^high^ (yellow arrows; intense expression) sensory neurons and satellite glial cells (positions of SGCs are indicated using white arrows).

The knowledge about the specific receptor for MANF was unknown for a long time. However, a recent study by Yagi et al., demonstrated that MANF binds to neuroplastin receptor for mediating its anti-inflammatory actions (Yagi et al., 2020). The expression of neuroplastin in PNS tissues has not been examined previously. Therefore, we examined the expression of neuroplastin in normal and injured (axotomized or *in vivo* primed) DRGs and found that it is expressed in both NF200^low^ and NF200^high^ sensory neurons (Fig. 8*A*). Importantly, satellite glial cells gained its expression after axotomy (Fig. 8*B*). We earlier observed that NF200^high^ neurons and satellite glial cells have low intrinsic expression of MANF, while NF200^low^ neurons express it at higher levels even after injury (Fig. 7*A*). Collectively, our observation suggests that MANF derived from NF200^low^ neurons may bind to neuroplastin receptors on NF200^high^ neurons and glial cells and activate them for nerve regeneration. These possibilities warrant additional investigations of MANF-neuroplastin axis in the PNS.

## Discussion

Despite active research in the past several decades, no drug therapy is available for the effective repair of injured peripheral nerves. While nerve reconstruction surgeries may be helpful for short distance regeneration, a combined approach of surgery and drug therapy is highly desired for long-distance regeneration and better functional recovery (López-Cebral et al., 2017). Here, by performing a comparative proteomics analysis of *in vitro* and *in vivo* primed DRGs, we were able to shortlist molecular candidates that are likely modulators of adult axon growth. We believe that this approach is more robust in identifying potential candidates for adult nerve regeneration since the candidates were derived from two independent growth-priming models.

The priming of DRGs preconditions adult neurons for regeneration, and hence the commonly upregulated proteins in our two models are likely promoters of axon growth. However, the opposite may also be true, such that the upregulated proteins may be growth inhibitory. For example, preconditioning of injured neurons also induces expression of growth inhibitors (Christie et al., 2014; Krishnan et al., 2016). Hence, follow-up studies of the commonly upregulated critical proteins are warranted. We were keen in initially studying MANF because of its previously reported neuroprotective, axon polarizing, and neurite outgrowth roles in CNS neurons (Tseng et al., 2017; Eesmaa et al., 2021). MANF was also shown to contribute to cell metabolism (Danilova et al., 2019), and inclined to this, our proteomics data revealed several molecular changes associated with cell metabolism in the primed DRGs. Additionally, we validated the expression of MANF predominantly in NF200^low^ sensory neurons, with very weak to no detectable endogenous levels in basal and injury activated glia. We also demonstrated the presence its receptor Neuroplastin, especially in NF200^high^ neurons and injury activated glia, suggesting that exogenous MANF may modify cellular signaling in these cells by binding to neuroplastin. Substantiating this argument, we demonstrated that exogenous MANF promotes the outgrowth parameters of NF200^high^ sensory neurons in culture. Efficient regeneration of large diameter NF200^high^ axons is critical for peripheral nerve repair because of them being a major population that is heavily myelinated. Therefore, the growth promoting effect of MANF in this population is promising to consider it as a potential nerve repair agent. MANF has been previously shown to regulate glial cell signaling (Walkowicz et al., 2017). Studies in the past has also showed that MANF is a secretory protein (Li-Na et al., 2017). Although we did not find its remarkable release from cultured neurons, its secretion from injured neurons *in vivo* cannot be ruled out, especially since its receptor is expressed in injury activated glia. Therefore, further validation of MANF in more rigorous peripheral nerve injury models *in vivo* is warranted. Overall, our proteomics exploration of primed DRGs identified previously unrecognized critical roles of MANF in the PNS.

We found common downregulation of several transport proteins in the DRGs after *in vitro* and *in vivo* priming. While their downregulation in the *in vitro* primed DRGs was not surprising, may be because of the lack of longer nerve connections to neuronal soma in this model requiring no major retrograde transport, such a downregulation in the *in vivo* DRGs was not expected as retrograde transport is critical for axotomy-driven nerve preconditioning (Ducommun Priest et al., 2019). However, the unique upregulation of the transport proteins dynamin-3, dynein 1 light intermediate chain 2, and KIF5A after *in vivo* priming indicates that these proteins may be critically required for the long-distance retrograde transport *in vivo*. We also found the upregulation of the transport protein importin subunit α−5 in the *in vivo* primed DRGs although our focused analysis considering selected biological processes did not capture this protein. The common downregulation of the neurite outgrowth facilitators such as contactin-1, CDK5, NCAM-1, and peroxisome modulators in the primed DRGs were unexpected, but indicate the presence of upstream growth decelerating signals in these DRGs. Supporting this argument, several previous studies demonstrated that growth inhibitory signals are active past neuronal injury and during the early regenerative reprogramming of adult neurons (Christie et al., 2014; Krishnan and Zochodne, 2015; Krishnan et al., 2016).

Overall, we identified 263 molecular candidates based on their differential expression in two growth priming models and tabulated their individual functions from the UniProt knowledgebase for further investigation. We also shortlisted most promising candidates among them based on their functional enrichments at the nerve growth-related biological processes. In addition, we identified a novel growth promoting role for MANF in the PNS along with the detection of its receptor neuroplastin in PNS tissues. This is a promising start and follow-up systematic screening of the shortlisted candidates in nerve regeneration models is warranted to explore the full potential of these candidates in improving adult nerve regeneration.

## Synthesis

Reviewing Editor: Eran Perlson, Tel Aviv University Decisions are customarily a result of the Reviewing Editor and the peer reviewers coming together and discussing their recommendations until a consensus is reached. When revisions are invited, a fact-based synthesis statement explaining their decision and outlining what is needed to prepare a revision will be listed below. The following reviewer(s) agreed to reveal their identity: Ella Doron Mandel.

The authors have addressed most of the concerns raised by the reviewers. However, there are some further clarifications and suggestions needed authors responses before publication.

The modifications to how the proteomics data are presented are still insufficient. There is not a sufficient description of the proteomics results in the result section, like how many peptides/protein groups were identified? How many of those are differential?

It will be good to add heatmaps for the protein expression levels or the fold changes between samples and controls, broken down by biological replication, to assess reproducibility. The proteomics data swould benefit to have one panel in the figure, such as a volcano plot clearly showing fold changes and corrected p-values.

In addition, although the complete GO terms enrichment analysis is now given in a supplementary table, it is suggested to:

1. Present it in a figure so the reader can quickly evaluate it without downloading and opening a support file.

2. P-values or other statistical values should be provided.

3. The top X pathways should be presented (ranked either by “strength” or p-value) so that the reader can evaluate what indeed are the top enriched pathways.
